# Design and rationale for the randomised, double-blinded, placebo-controlled Liraglutide to Improve corONary haemodynamics during Exercise streSS (LIONESS) crossover study

**DOI:** 10.1186/s12933-015-0193-4

**Published:** 2015-02-19

**Authors:** Aung Myat, Satpal Arri, Deepak L Bhatt, Bernard J Gersh, Simon R Redwood, Michael S Marber

**Affiliations:** King’s College London British Heart Foundation Centre of Research Excellence, The Rayne Institute, Cardiovascular Division, St Thomas’ Hospital, Westminster Bridge Road, London, SE1 7EH UK; Brigham and Women’s Hospital Heart & Vascular Centre and Harvard Medical School, Boston, MA 02115 USA; Division of Cardiovascular Diseases, Mayo Clinic College of Medicine, 200 First Street SW, Rochester, MN 55905 USA

**Keywords:** Glucagon-like peptide-1 receptor agonist, Incretin hormone, Liraglutide, Chronic stable angina, Exercise-induced ischaemia, Type 2 diabetes mellitus

## Abstract

**Background:**

Glucagon-like peptide-1 is an incretin hormone essential for normal human glucose homeostasis. Expression of the glucagon-like peptide-1 receptor in the myocardium has fuelled growing interest in the direct and indirect cardiovascular effects of native glucagon-like peptide-1, its degradation product glucagon-like peptide-1(9-36), and the synthetic glucagon-like peptide-1 receptor agonists. Preclinical studies have demonstrated cardioprotective actions of all three compounds in the setting of experimental myocardial infarction and left ventricular systolic dysfunction. This has led to Phase 2 trials of native glucagon-like peptide-1 and incretin-based therapies in humans with and without Type 2 diabetes mellitus. These studies have demonstrated the ability of glucagon-like peptide-1, independent of glycaemic control, to positively modulate the metabolic and haemodynamic parameters of individuals with coronary artery disease and left ventricular systolic dysfunction. We aim to add to this growing body of evidence by studying the effect of chronic glucagon-like peptide-1 receptor activation on exercise-induced ischaemia in patients with chronic stable angina managed conservatively or awaiting revascularisation. The hypothesis being liraglutide, a subcutaneously injectable glucagon-like peptide-1 receptor agonist, is able to improve exercise haemodynamics in patients with obstructive coronary artery disease when compared with saline placebo.

**Methods and design:**

The Liraglutide to Improve corONary haemodynamics during Exercise streSS (LIONESS) trial is an investigator-initiated single-centre randomised double-blinded placebo-controlled crossover proof-of-principle physiological study. Primary endpoints are change in rate pressure product at 0.1 mV ST-segment depression and change in degree of ST-segment depression at peak exercise during sequential exercise tolerance testing performed over a 6-week study period in which 26 patients will be randomised to either liraglutide or saline with crossover to the opposing regimen at week 3.

**Discussion:**

The study will be conducted in accordance with the principles of Good Clinical Practice and the Declaration of Helsinki. The local Research Ethics Committee and Medicines and Healthcare Products Regulatory Agency have approved the study.

**Trial registration:**

National Institute of Health Research Clinical Research Network (NIHR CRN) Portfolio ID 11112 and ClinicalTrials.gov Identifier NCT02315001.

## Background

The incretin concept was borne from the observation that an oral load of glucose can provoke a two to three times more potent insulinotropic stimulus than an isoglycaemic intravenous glucose infusion [1,2]. This effect has been attributed to the action of incretin (INtestinal seCRETion of INsulin) hormones, which constitute part of the glucagon superfamily. Glucose-dependent insulinotropic polypeptide (GIP), a 42-amino acid peptide made by duodenal and jejunal enteroendocrine K cells in the proximal small bowel, was the first incretin hormone to be isolated from purified porcine intestinal extracts [3]. More than a decade later, glucagon-like peptide-1 (GLP-1) was identified, a 30-amino acid cleavage product of proglucagon, synthesised predominantly by the enteroendocrine L cells of the distal ileum and colon [4,5]. Despite their site of production, plasma levels of GIP and GLP-1 rise within minutes of enteral nutrition, indicating a combined neural and endocrine signalling axis prompting their secretion [6]. Together, GIP and GLP-1 are fundamental to maintaining normal glucose homeostasis in man. They contribute almost equally to, and have an additive effect on, enhancing glucose-dependent insulin exocytosis after meal ingestion, although GLP-1-mediated effects appear to predominate at higher glucose levels [7].

There is a defective incretin effect in Type 2 diabetes mellitus (T2DM) [8]. Levels of GIP can increase after enteral nutrition but the ability to potentiate postprandial insulin secretion is significantly impaired [9,10]. In contrast, meal-stimulated levels of GLP-1 are severely depressed. A continuous infusion of exogenous GLP-1 can, however, result in a near normal insulin-mediated response to an oral glucose load, suggesting preservation of insulinotropic activity [11-14]. Furthermore the risk of hypoglycaemia with GLP-1 is minimal, as both its stimulatory effect on insulin secretion and its inhibitory action on glucagon release switch off when ambient glucose levels are <4 mmol/L [5,14,15]. Despite these properties, the pharmaco-therapeutic utility of native GLP-1 in T2DM is profoundly limited by its rapid inactivation by the enzyme dipeptidyl dipeptidase-4 (DPP-4), which cleaves 2 amino terminal peptides from GLP-1(7-36) to form the GLP-1(9-36) metabolite [5]. GLP-1 has a half-life of <2 minutes which means only 10-20% of total plasma GLP-1 is biologically active [16]. Novel treatment strategies for T2DM, based on the incretin effect, have been developed to overcome this endogenous cul-de-sac. Subcutaneously injectable GLP-1 receptor (GLP-1R) agonists (DPP-4 mediated degradation-resistant peptides with improved pharmacokinetics that act via the human GLP-1R) and oral DPP-4 inhibitors (small molecules with clinically useful oral bioavailability that shield the endogenous peptide from DPP-4 metabolism and thereby enhance its innate insulinotropic activity) are now licensed and have become part of the standard of care for the management of T2DM [17-19]. In essence GLP-1R agonists provide pharmacologic levels of a GLP-1R stimulus whereas DPP-4 inhibitors preserve physiologic levels of endogenous GLP-1 [6].

The GLP-1R is a heptahelical G-protein coupled receptor found widely in several extra-pancreatic tissue beds, including lung, kidney, central, enteric and peripheral nervous systems, lymphocytes, smooth muscle cells and atrial cardiomyocytes [20-23]. It is this expression of the GLP-1R in the myocardium, and the overall ubiquity of the receptor, that has fuelled growing interest in the direct and indirect cardiovascular (CV) effects of native GLP-1 [24,25], its degradation product GLP-1(9-36) [26,27], and the degradation-resistant GLP-1R agonists [28-30]. Indeed knockout mice lacking the GLP-1R were shown to have a lower resting heart rate, elevated left ventricular (LV) end diastolic pressure and increased LV wall thickness compared to wild type controls [31]. These mice also displayed depressed LV contractility and impaired diastolic function after insulin administration; all suggesting a pivotal role for the GLP-1R in maintaining normal cardiac structure and function, in mice at least [31,32]. It is also important to note that whilst native GLP-1 may exert a regulatory role on CV biology through its interaction with the GLP-1R plus its independently cardioactive metabolite GLP-1(9-36), synthetic GLP-1R agonists are shielded from DPP-4 breakdown and therefore act predominantly via their action on the classical GLP-1R alone [5,20]. Nevertheless, several preclinical studies have demonstrated cardioprotective actions of all three compounds in rodent, canine and porcine hearts in the context of ischaemia/reperfusion (I/R) injury after experimental myocardial infarction (MI) and LV systolic dysfunction [24,26,27,29,30,33-36]. The growing weight of evidence from these animal models has led to several Phase 2 trials of native GLP-1 and incretin-based therapies in humans with and without T2DM (Table 1). Although hypothesis-generating at best, these small, often single-centre, studies have demonstrated the ability of GLP-1, independent of its effect on glycaemic control, to positively modulate the metabolic and haemodynamic parameters of individuals with CAD and cardiomyopathy (Table 1) [37-45].

**Table 1:** **Evidence for GLP-1-mediated cardioprotection in humans**

**Author and year published**	**Pathologic substrate**	**Agent**	**Control**	**Hypothesis/Question**	**Pertinent findings**	**Potential Limitations**
Nikolaidis et al. 2004 [37]	Myocardial ischaemia/reperfusion injury	GLP-1 (N=10)	Standard therapy post PPCI (N=11)	Can a 72-hour infusion of GLP-1 improve global and regional LV function for post infarct myocardial dysfunction following successful PPCI?	• GLP-1 therapy improved global LVEF (p<0.01)	• Small, single-centre, nonrandomized pilot study
					• GLP-1 improved regional (p<0.001) and global (p<0.001) WMSI	• Truncated 4-day follow-up window does not allow for extrapolation of results
					• Improvements seen in diabetics and non-diabetics and after anterior and non-anterior MI	
					• GLP-1 reduced hospital stay significantly (p<0.02)	
Sokos et al. 2006 [38]	Dilated Cardiomyopathy	GLP-1 (n=12)	Maximum standard therapy (n=9)	Can a 5-week subcutaneous infusion of GLP-1 improve both LVEF and functional capacity?	• LVEF improved significantly in the GLP-1 arm ((p<0.001) and was unchanged in the control arm	• Small, single-centre, open-label, nonrandomised study
						• Type I diabetics excluded but not Type II – potential source of confounding and increased incidence of hypoglycaemia
					• 6MWT distance improved significantly in the GLP-1 arm (p<0.001)	
					• Quality of life improved significantly with GLP-1 (p<0.001)	• No mention of exact infusion volume – essential in a heart failure cohort
					• Functional improvements seen in diabetics and non-diabetics	
Sokos et al. 2007 [39]	CABG surgery	GLP-1 (n=12)	Standard therapy (n=12)	Can peri- and postoperative GLP-1 administration improve haemodynamic recovery after CABG surgery?	• No difference in LVEF or cardiac index between the groups	• Small numbers despite randomisation
					• Control group required greater use of inotropic and vasoactive infusions	• Hypothesis-generating
					• More frequent arrhythmias seen in control group	
Halbirk et al. 2010 [40]	Ischaemic cardiomyopathy	GLP-1 (n=10 crossover)	Saline (n=10 crossover)	GLP-1 can improve cardiac function and exercise capacity in non-diabetic patients with heart failure.	• Cardiac index and LVEF remained unchanged	• Small, single-centre study
					• BNP levels remained unchanged	• Active intervention with a 48-hour GLP-1 infusion may have been too short to mediate any improvement in cardiovascular indices
					• Hypoglycaemic events related to GLP-1 treatment were seen in 8 patients	
						• Trial protocol only completed in 75% of patients
Read et al. 2010 [41]	Myocardial ischaemia (mediated by dobutamine stress)	Sitagliptin (n=14 crossover)	Placebo (n=14 crossover)	Increased availability of endogenous GLP-1 through DPP-4 inhibition will protect the heart against postischaemic LV dysfunction.	• Greater increase in myocardial performance after sitagliptin at peak stress (p=0.0001)	• Small study sample
					• Myocardial stunning seen in controls after dobutamine stress whereas sitagliptin maintained LV function	• Hypothesis-generating
					• Sitagliptin had a greater beneficial effect on ischaemic vs. nonischaemic LV segments	
Read et al. 2011 [42]	Myocardial ischaemia/reperfusion injury	GLP-1 (n=10)	Saline (n=10)	Can GLP-1 protect the heart against ischaemic dysfunction associated with serial 1-minute coronary balloon occlusions during PCI and mitigate myocardial stunning?	• GLP-1 infusion improved recovery of LV systolic and diastolic function at 30 minutes post 1-minute coronary balloon occlusion compared with control (p=0.02)	• Study too small to assess any clinical endpoints
						• Coronary flow not assessed
					• GLP-1 infusion reduced LV dysfunction after a second 1-minute coronary balloon occlusion compared with control (p=0.01)	• Hypothesis-generating
Read et al. 2012 [43]	Myocardial ischaemia (mediated by dobutamine stress)	GLP-1 (n=14 crossover)	Saline (n=14 crossover)	Can GLP-1 protect the heart from ischaemic LV dysfunction and improve myocardial response to dobutamine stress?	• Greater increase in LVEF at peak stress during GLP-1 infusion	• Small study sample
					• No myocardial stunning seen during GLP-1 infusion	• Study not powered to examine clinical end points
					• GLP-1 improved myocardial performance specifically in LV segments subtended by a stenosed vessel and did not in segments receiving an unobstructed blood supply	
Lønborg et al. 2012 [44]	Myocardial I/R injury	Exenatide (n=85)	Saline (n=87)	Can exenatide protect against reperfusion injury in STEMI patients following PPCI?	• Significantly greater myocardial salvage index in the exenatide group (p=0.003) post PPCI	• LVEF after 90 days was not significantly different between the two groups
					• Patients in the exenatide group developed significantly smaller infarcts for an equivalent area at risk (p=0.011)	• Study cohort too small to detect a difference in 30-day clinical events
McCormick et al. 2014 [45]	Myocardial ischaemia (mediated by dobutamine stress)	Sitagliptin (taken for 4 weeks) (n=19)	Standard oral hypoglycaemic agents (n=19)	Can chronic DPP-4 inhibition with sitagliptin protect the heart from ischaemic LV dysfunction and improve myocardial response to demand ischaemia during dobutamine stress in Type 2 diabetes patients with CAD	• No difference in the rate pressure products at baseline, peak stress, or recovery between the sitagliptin and control scans	• Small study sample
						• Cannot exclude degree of variation in individual response to dobutamine during 2 consecutive stress echocardiograms separated by a number of weeks
					• At peak stress there was a greater increase in global ejection fraction following sitagliptin therapy (p<0.0001)	
					• At peak stress sitagliptin enhanced regional LV function – seen predominantly in ischaemic segments (p=0.001) whereas there was no effect in non-ischaemic segments (p=0.87)	• CAD defined by the presence of a single proximal stenosis >50% in at least 1 epicardial coronary artery – some might argue this level of obstruction would not be haemodynamically significant

Key: GLP-1 = glucagon-like peptide-1; PPCI = primary percutaneous coronary intervention; LVEF = left ventricular ejection fraction; 6MWT = 6-minute walk test; WMSI = wall motion score index; BNP = brain natriuretic peptide; CABG = coronary artery bypass grafting; STEMI = ST-elevation myocardial infarction; PCI = percutaneous coronary intervention; DPP-4 = dipeptidyl dipeptidase-4; CAD = coronary artery disease.

Our aim is to add to this growing body of evidence by characterising, for the first time, the effect of chronic GLP-1R activation on haemodynamics and exercise-induced ischaemia in a cohort of patients with chronic stable angina managed conservatively or awaiting revascularisation. Rather than study I/R injury following coronary occlusion, we will determine whether the acute anti-ischaemic properties of GLP-1 translate into an anti-anginal action on exertion. Liraglutide (trade name Victoza manufactured by NovoNordisk, Bagsvaerd, Denmark), a synthetic GLP-1R agonist that shares 97% structural homology with native GLP-1, has a half-life of 10–14 hours. It binds to albumin in the circulation, protecting it from DPP-4 degradation and preventing renal elimination. For these reasons liraglutide is a useful surrogate for studying chronic GLP-1R activation in humans. Moreover the need for a continuous GLP-1 infusion is now replaced by a once daily subcutaneous injection, which should help to maximise patient compliance and preclude the risk of fluid overload in those with established coronary heart disease. The relatively small risk of hypoglycaemia associated with liraglutide serves to further support it being used to study the effects of GLP-1 beyond glycaemic control in both diabetics and non-diabetics. Indeed, a trial investigating the treatment of obesity demonstrated significantly greater weight loss achieved with liraglutide versus orlistat or placebo and an absence of hypoglycaemic episodes in a cohort of non-diabetics [46]. Our centre has developed reproducible exercise protocols for the study of angina [47-50], and it is from these that the current trial protocol has been derived.

### Aims and objectives

The objective of the present protocol is to evaluate the physiological basis of chronic GLP-1R occupancy on exercise haemodynamics, as manifest by specific electrophysiological parameters, in those patients with known chronic stable angina, a significant coronary stenosis in at least one major epicardial vessel confirmed by coronary angiography and evidence of reversible myocardial ischaemia seen on exercise tolerance testing. The hypothesis being liraglutide, a subcutaneously injectable synthetic analogue of endogenous GLP-1, is able to improve exercise haemodynamics in a cohort of patients with obstructive coronary artery disease (CAD) when compared with saline placebo. Primary and secondary outcome measures are listed in Tables 2 and 3 respectively.

**Table 2:** **Primary outcome measures**

•	Change in rate pressure product at 0.1 mV ST-segment depression during sequential exercise tolerance testing performed over a 6-week study period
•	Change in degree of ST-segment depression at peak exercise during sequential exercise tolerance testing performed over a 6-week study period

**Table 3:** **Secondary outcome measures**

•	Change in total exercise time during sequential exercise tolerance testing performed over a 6-week study period
•	Change in time to 0.1 mV ST-segment depression during sequential exercise tolerance testing performed over a 6-week study period
•	Change in time to maximum ST-segment depression during sequential exercise tolerance testing performed over a 6-week study period
•	Change in recovery time to 0.05 mV ST-segment depression during sequential exercise tolerance testing performed over a 6-week study period
•	Evidence of hypoglycaemia through twice-daily home blood glucose monitoring and once-weekly random serum glucose measurements
•	Evidence of renal dysfunction through once-weekly monitoring of serum creatinine, electrolytes and estimated glomerular filtration rate
•	Evidence of acute pancreatitis through once weekly monitoring of serum amylase alongside telephone and once-weekly face-to-face study visits

## Methods and design

### Study design

The **L**iraglutide to **I**mprove cor**ON**ary haemodynamics during **E**xercise stre**SS** (LIONESS) (NIHR CRN Study ID 11112 and clinicaltrials.gov NCT02315001) trial is an investigator-initiated single-centre randomised double-blinded placebo-controlled crossover proof-of-principle physiological study. Patients will be enrolled according to the inclusion and exclusion criteria listed in Tables 4 and 5 respectively.

**Table 4:** **Inclusion criteria**

•	Men and women aged 18-80
•	Patients must be able to walk confidently on an exercise treadmill
•	Patient must have a recent abnormal exercise tolerance test demonstrating >0.1 mV of planar or down-sloping ST-segment depression
•	Patients must have angiographic evidence of a >70% stenosis in a main epicardial coronary artery, with or without coronary stenoses elsewhere
•	Patients must have a normal resting electrocardiogram in sinus rhythm without bundle branch aberration or other conduction disturbance
•	Patients must have preserved left ventricular systolic function (ejection fraction ≥40%)

**Table 5:** **Exclusion criteria**

•	An abnormal resting electrocardiogram including atrial fibrillation, bundle brunch aberration or other conduction disturbance
•	Pre-existing significant left ventricular systolic dysfunction (ejection fraction <40%)
•	Pre-existing ischaemic or non-ischaemic cardiomyopathy
•	Pre-existing haemodynamically significant valvular heart disease
•	Inability to safely negotiate an exercise treadmill
•	Patients with Type 1 diabetes mellitus
•	Patients with Type 2 diabetes mellitus taking oral and/or subcutaneous anti-diabetic therapy
•	Patients with a personal or family history of medullary thyroid carcinoma
•	Patients with Multiple Endocrine Neoplasia syndrome type 2
•	Patients with acute renal failure or deteriorating renal function

**Figure 1:**
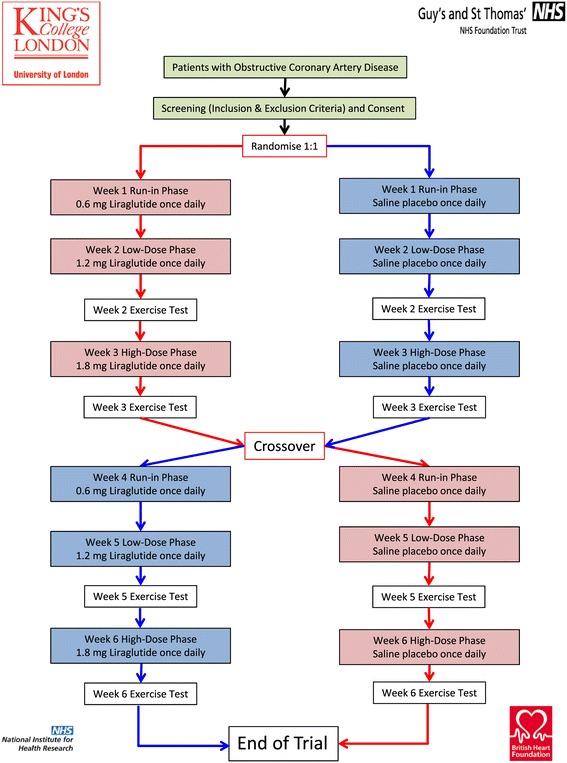
**The LIONESS Trial study design.**

Both investigator and patient will be blinded to the study drug. Patients will be randomised to enter a GLP-1 treatment arm or matched-volume saline placebo arm. Following a 1-week run-in phase with 0.6 mg liraglutide followed by a 1-week course of 1.2 mg liraglutide, patients in the active intervention arm will have their first exercise tolerance test (ETT) at the end of Week 2. Patients will then be up-titrated to high-dose 1.8 mg liraglutide for another week before performing a Week 3 ETT. With a stepwise increase in liraglutide therapy over a 3-week period we hope to observe a dose–response effect on exercise haemodynamics. An initial 1-week run-in phase of once daily 0.6 mg liraglutide should also allow for improved gastrointestinal tolerability. Whilst in the placebo arm, patients will have matched-volume saline injections for the first two weeks before the Week 2 ETT and then another week of saline injections before the Week 3 ETT. Patients will then crossover at this stage so that those in the treatment arm cross to the placebo arm and vice versa (please refer to Figure 1).

Criteria for terminating an exercise stress test will be: 1) physical exhaustion, 2) severe chest pain, 3) attaining maximal age-related heart rate, 4) ST-segment depression (STD) >0.4 mV, or 5) haemodynamically-significant dysrhythmia.

### Sample selection

Potential trial participants fulfilling the inclusion criteria (Table 4) and who consent will be recruited consecutively from the following groups:

- Patients with known obstructive CAD on angiography and a previously abnormal exercise test, but who are currently stable on conservative medical management, will be enrolled from general cardiology outpatient clinic.
- Patients with known obstructive CAD on angiography and evidence of reversible myocardial ischaemia on exercise testing, awaiting elective percutaneous coronary intervention (PCI) or coronary artery bypass graft (CABG) surgery.

### Sample size calculation

The host department has shown that the time taken to 0.1 mV STD (368 ± 34 → 418 ± 36 seconds) and the rate pressure product at 0.1 mV STD (20500 ± 755 → 21907 ± 764 mmHg/min) is significantly increased during the second of two serial exercise tests separated by 15 minutes in chronic CAD patients with known left anterior descending artery stenoses [47,48]. This effect is attributed to warm-up angina, which is thought to augment the innate resistance of the myocardium to an ischaemic insult [51,52]. We would expect the administration of Liraglutide to mimic the beneficial cardioprotective effects of warm-up angina. A sample size of 26 patients randomised in a 1:1 fashion to each treatment arm (taking into consideration a >10% drop-out rate) followed by crossover would have 90% power to detect a difference between the means of approximately 15% (2-sided α = 0.05).

### Randomisation procedure

All randomisations will be performed through a computer-generated randomisation program (www.sealedenvelope.com) in a 1:1 randomisation ratio utilising a study-specific ID assigned to each enrolled patient. Treatment allocation can be unblinded in case of an emergency or serious adverse event if deemed necessary by the investigator.

### Study agent and placebo comparator

Liraglutide will be supplied to trial recruits in pre-filled single-use plastic syringes ready for once daily subcutaneous injection at doses of 0.6 mg, 1.2 mg and 1.8 mg. Matched-volume saline placebo will also be supplied in identical pre-filled syringes for subcutaneous injection. Study medication will be produced and dispensed by the Aseptic Services Unit of St Thomas’ Hospital Pharmacy as a set of 7 separate disposable syringes at the beginning of each study week. Patients will be asked to store these in a refrigerator and to dispose of their used syringes in a sharps bin. The sealed bin will be brought to the next study visit to check for compliance with the study agents. Patients will be advised to administer their study agent at approximately the same time each day to reinforce compliance. The recommended injection site will be the abdomen, and that injection site around the abdomen should be varied from day to day.

### Concomitant medication

Study participants will have any heart rate-limiting drugs cautiously withdrawn prior to commencing the 6-week trial protocol. These include beta-blockers, rate-limiting calcium antagonists (e.g., diltiazem and verapamil), and ivabradine to ensure a normal chronotropic response to exercise stress testing. Long-acting oral nitrates and nicorandil will also be stopped to avoid any potential interference with haemodynamic physiology. Patients will be allowed to continue their antiplatelet therapy (e.g., aspirin, clopidogrel, prasugrel, and/or ticagrelor), ACE-inhibitors, angiotensin receptor blockers, and statins. Patients will also continue their sublingual glyceryl trinitrate (GTN) spray or buccal GTN to relieve breakthrough angina. Those patients experiencing poor control of pre-existing hypertension following the cessation of the drugs listed previously will be prescribed alternative antihypertensive medication at the discretion of the investigator, or have their current medication they can continue up-titrated, and monitored closely for response.

## Study visits and procedures

### Screening Visit

Once a patient is deemed to have adequately fulfilled the inclusion and exclusion criteria, informed consent will be obtained. Part of the inclusion criteria includes the demonstration of reversible ischaemia during an ETT. If a recent ETT is not available, the patient will be asked to perform an ETT (free of any heart rate-limiting medication for the past 48 hours) at the screening visit. The result of this ETT will not be used in the final data analysis but will be captured as part of the screening process. Those patients ineligible for trial participation will be registered, and the reason for screening failure will be recorded. Patients entering the trial will receive a tutorial on home blood glucose monitoring and the signs and symptoms of hypoglycaemia. A safety information card will be issued. A full medical history and clinical examination will be performed. Blood tests will be taken (Table 6). Medications that need to be withheld will be identified at this visit and withdrawn cautiously (see Table 7). Those patients successfully recruited will be randomised at this stage.

**Table 6:** **Laboratory tests**

**The following blood tests will be taken at each study visit (Table** **7** **) unless otherwise stated:**	
•	Full blood count
•	Creatinine and electrolytes
•	Estimated glomerular filtration rate
•	Liver function tests
•	Amylase
•	Corrected calcium
•	Random blood glucose
•	HBA_1c_ (Week 3 and Week 6 only)
•	Random lipid profile (serum total cholesterol, low-density lipoprotein cholesterol, high-density lipoprotein cholesterol, and triglyceride) (Week 3 and Week 6 only)

**Table 7:** **LIONESS trial flowchart of visits and procedures**

	**Screening visit**	**Commence trial (Week 0)**	**Study visit (Week 1)**	**Exercise test 1 (Week 2)**	**Exercise test 2 (Week 3)**	**Study visit (Week 4)**	**Exercise test 3 (Week 5)**	**Exercise test 4 (Week 6)**
Obtain informed consent, issue safety information card and patient diary	Yes	-	-	-	-	-	-	-
Randomisation to study agents	Yes	Perform first self-administration of study agent	-	-	-	-	-	-
Issue 7-day course of blinded study agent	-	Yes	Yes	Yes	Yes	Yes	Yes	Stop all study medication
Check study drug compliance	-	-	Yes	Yes	Yes	Yes	Yes	Yes
Withdraw protocol-mandated pre-existing patient medication	Tutorial and commence withdrawal	Complete withdrawal	-	-	-	-	-	Recommence pre-existing patient medication
Home Blood Glucose Monitoring	Tutorial and issue equipment	-	Record data from patient diary	Record data from patient diary	Record data from patient diary	Record data from patient diary	Record data from patient diary	Record data from patient diary
Physical examination	Yes	Yes	Yes	Yes	Yes	Yes	Yes	Yes
Height measurement	Yes	-	-	-	-	-	-	-
Weight measurement	Yes	Yes	Yes	Yes	Yes	Yes	Yes	Yes
Blood pressure measurement	Yes	Yes	Yes	Yes	Yes	Yes	Yes	Yes
Electrocardiogram	Yes	-	-	-	-	-	-	-
Pre-specified blood tests	Yes	Yes	Yes	Yes	Yes	Yes	Yes	Yes
Perform supervised exercise tolerance test (ETT)	Yes (if recent ETT unavailable)	-	-	Yes	Yes	-	Yes	Yes

### Trial visits (week 0 to week 6)

Trial participants will perform their first self-administration of study agent and first blood glucose measurement, which will be recorded in their patient diary, at trial commencement (Week 0). Both will be supervised by an investigator. Recruited patients will attend a total of 7 study visits and have procedures performed according to the schedule detailed in Table 7. Patients will be asked to record twice daily home blood glucose measurements at approximately the same time am and pm. Patients will have blood tests performed at every study visit (Table 6).

### Expected duration of the study

From the point of administration of study drug on day 1 the total length of the study will be 6 weeks. There will be no follow-up of patients after completion of the 6-week trial protocol, at which point study drugs will be terminated and patients will have their pre-study medication recommenced immediately thereafter.

### Procedures for recording and reporting adverse events

All study participants will be issued a patient diary in which they can record any adverse events, side effects, injection site reactions and angina burden. This diary will be inspected at every study visit. Patients will also be able to contact the Main Patient Liaison at all times for general enquiries and advice regarding any signs and symptoms they are experiencing pertaining to the study. The primary care physicians of all study participants will be issued with an information sheet regarding the potential side effects of the GLP-1R agonist Liraglutide and the medication that a patient may have had discontinued prior to commencing the trial. The importance of informing the Main Patient Liaison if any adverse events were to occur will be stressed.

### Withdrawal of subjects

Participants will have the right to withdraw from the study at any time for any reason. Should a patient decide to withdraw from the study, all efforts will be made to report the reason for withdrawal as thoroughly as possible. The investigator also has the right to withdraw patients from the study in the event of inter-current illness, adverse events or serious adverse events, protocol violations, administrative reasons or other reasons.

The study drug will be discontinued and the participant withdrawn from the study if:

- the participant misses 2 consecutive courses of treatment;
- the participant decides they no longer wish to continue;
- if the participant experiences an intolerable degree of angina as a result of withdrawing their pre-study heart rate-limiting or anti-anginal medications;
- if the participant experiences significant side effects from their study medication, in particular excessive or intolerable nausea, vomiting or diarrhoea; or
- the investigator recommends it.

The Sponsor or Chief Investigator may prematurely discontinue the study on the basis of new safety information or for other reasons given by the Study Steering Committee or Research Ethics Committee concerned.

### Data management and analysis

Data will be collected prospectively using a customised paper Case Report Form (CRF). Investigators will record adverse events, injection site reactions, angina episodes and clinical and laboratory parameters on the CRF at each study visit.

The fundamental principle underpinning the design of this trial is that the 2-week period between the Week 3 ETT and the Week 5 ETT (see Figure 1), in those patients randomised to the active liraglutide intervention first, should be sufficient to allow adequate washout of the drug and therefore minimise the impact of a potential carryover effect. Given that the half-life of liraglutide is 10–14 hours, this 2-week washout period far exceeds the generally accepted mark of ≥5 half-lives. A washout period for those randomised to the placebo arm first would not apply per se. A preliminary test, such as the Wilcoxon rank sum test, will be applied to ensure there is no carryover effect. Once this has been established, standard two-sample t-tests for independent samples using the intra-individual differences between the outcomes in both study periods as the raw data will be utilised [53].

A multiple regression model will be used to compare treatment, visit number, and patient, using least squares. Linear regression will be used to study the correlation between outcome parameters and baseline patient characteristics. A p value <0.05 will be considered statistically significant.

## Discussion

The study will be conducted in compliance with the principles of the Declaration of Helsinki (2008), the principles of Good Clinical Practice and in accordance with all applicable requirements laid down by the Co-Sponsors of the study, namely: King’s College London and Guy’s and St Thomas’ NHS Foundation Trust. The trial protocol has received ethical approval from the National Research Ethics Service (NRES) Committee London – Westminster (Research Ethics Committee reference 11/LO/1564) of the United Kingdom. Annual progress and safety reports and a final report at conclusion of the study will be submitted to the Co-Sponsors and the Research Ethics Committee. The Medicines and Healthcare Products Regulatory Agency (MHRA) has also approved the study.

This trial has been funded by the British Heart Foundation via a Clinical Research Training Fellowship awarded to Dr Aung Myat (grant number FS/11/70/28917) and is supported by the Department of Health via the National Institute of Health Research comprehensive Biomedical Research Centre award to Guy’s and St Thomas’ NHS Foundation Trust in partnership with King’s College London and King’s College Hospital NHS Foundation Trust.

The LIONESS study is a single-centre trial based in the United Kingdom. The trial has, therefore, been registered with our national trials database via the National Institute of Health Research Clinical Research Network (NIHR CRN) portfolio (Study ID 11112) since May 4th 2012. The NIHR CRN trials database is accessible to the public at no charge, open to all prospective registrants, managed by a not-for-profit organization, has a mechanism to ensure the validity of the registration data, and is electronically searchable. The study has also been registered on ClinicalTrials.gov as of November 10th 2014 (NCT02315001). There have been minor amendments to the study protocol since first registration and as such the first patient was recruited in January 2014. The study remains actively recruiting as of February 2015.

It is intended that positive or negative study results will be reported and disseminated at international conferences and in an international peer-reviewed scientific journal.
